# Anticipation and prevention of real risks of virtual environments in psychiatry

**DOI:** 10.1038/s41746-026-02348-4

**Published:** 2026-01-30

**Authors:** Maria Marloth, Celia Deane-Drummond, Philipp Kellmeyer, Marc Erich Latoschik, Jennifer A. Chandler, Gerben Meynen, Kai Vogeley

**Affiliations:** 1 Department of Psychiatry and Psychotherapy, Faculty of Medicine and University Hospital of Cologne, Cologne, Germany; 2 Laudato Si’ Research Institute, Campion Hall, and Faculty of Theology and Religion, University of Oxford, Oxford, United Kingdom; 3 School of Business Informatics and Mathematics, University of Mannheim, Mannheim, Germany; 4 Department of Neurosurgery, University of Freiburg - Medical Center, Freiburg im Breisgau, Germany; 5 Human-Computer Interaction Group, Faculty of Mathematics and Computer Science, Wuerzburg University, Wuerzburg, Germany; 6 Faculty of Law and Faculty of Medicine (by cross-appointment), University of Ottawa, Bruyère Research Institute, Ottawa, Ontario, Canada; 7 Department of Philosophy, Faculty of Humanities, VU University Amsterdam & Willem Pompe Institute for Criminal Law and Criminology, Department of Law, Utrecht University, Utrecht, Netherlands; 8 Institute for Neuroscience and Medicine, Cognitive Neuroscience (INM-3), Research Centre Juelich, Juelich, Germany

**Keywords:** Medical ethics, Psychology

## Abstract

Extended reality (=XR) provides promising opportunities for psychiatry in the future. However, psychiatric patients appear to be particularly vulnerable to virtual exposure. With a focus on virtual embodiment and virtual social interaction, we therefore, 1. describe the specific risks of virtual exposures, 2. discuss them in relation to specific psychopathological symptoms and 3. outline initial strategies that enable safe exposure with a strong emphasis on participatory designs.

## Introduction

Extended Reality (=XR) describes different computer-mediated environments along the virtuality continuum^1^, including Virtual Reality (VR), Mixed Reality (MR), and Augmented Reality (AR). Virtual Reality (=VR) denotes one end of this spectrum where the perceived environments purely consist of synthesized (computer-generated) content. This content can mimic the real physical world (but does not have to) and can also produce artificial environments.

The capacity to create entire experiences that are both safe and controllable makes the use of VR in medicine, and specifically in psychiatry, particularly interesting. Diagnostic and/or therapeutic interventions are being tested and developed for almost every clinical condition in psychiatry^2^. The initial enthusiasm that VR can offer patients replicas of reality, for example for the purpose of Virtual Reality exposure therapy (=VRET), is now being replaced by the growing awareness that the boundaries of reality can be even transcended in XR, and therapy options become possible which were not available without XR, for instance, the introduction of persons behind hallucinated voices (such as in the context of AVATAR therapy)^3,4^.

Despite these developments, XR in clinical practice has been implemented only in a very limited way. However, in view of the rapidly growing number of application ideas and studies, it seems only a matter of time before XR becomes an integral part of the treatment of patients not only in general but also in forensic psychiatry services^5^.

Such anticipatory evaluation is critical in psychiatry, where vulnerability of patients intersects with questions of agency, autonomy, and trust. In the case of XR applications in medicine, the need for this advancement was formulated by *The Lancet* as early as 1991^6^. Since then, various authors have argued in favor of formulating ethical guidelines for the application of VR in a general medical context, or, more specifically in a psychiatric context^7–9^. A recent publication summarized recommendations for the use of XR in older patients. These practical considerations are intended to serve as a basis for future evidence-based guidelines^10^. A nuanced understanding of risks and vulnerabilities is required for upholding the principle of *non-maleficence* (“*first, do no harm*”, from the Latin *primum non nocere*), which, since the Hippocratic Oath, has been one of the central tenets of biomedical ethics^11^. In medical practice, it refers primarily to the *weighing* of individual risks to which a patient is exposed by specific interventions and the extent to which such actions are justifiable in terms of potential benefits for the patient – as embodied by the ethical principle of *beneficence*. Careful and balanced evaluation of both the potential risks and benefits of any therapeutic technologies is required. This is especially true for novel therapies, for which the various possible biases need to be identified and considered^12^. An optimistic bias, termed *progress bias*, may lead to over-valuation of novelty^13^. Conversely, *status quo bias* is a well-documented psychological phenomenon, including in medical decision-making, leading to over-valuation of existing approaches^14^. These two general forms of biases are associated with under- and over-estimation of harms and risks associated with any novel technology, respectively.

Beyond these general effects, a more detailed examination of XR applications in psychiatry reveals specific potential benefits, they comprise 1. completely new approaches that are only possible because substantially different experiences can be made in virtual environments, 2. easier access also for people with disabilities, 3. better availability so that virtual psychotherapeutic offers can already be made available if personal therapeutic appointments cannot be guaranteed due to shortages, 4. augmentative use of VR approaches as a supplement to classical therapies. Because we are convinced that future XR therapies hold significant potential benefits for psychiatric patients, our aim is not to discourage innovation but to clarify the ethical boundaries necessary for its responsible implementation.

### Narrative review approach

This narrative review explores the complex relationship between the vulnerabilities of psychiatric patients and the risks of VR applications. It helps to map conceptual linkages, clarify areas of risk, and identify gaps in understanding. The review is based on publications addressing both the ethical aspects of using XR in psychiatry, as well as studies on XR applications in psychiatric and medical contexts from clinical and technological perspectives. Many of the risks described in ethical literature are currently anticipated as potential possibilities. While empirical data on psychiatric XR use remain limited, our approach follows the logic of anticipatory ethics that identify potential domains of harm and moral tension before the widespread implementation of new technologies. This distinction between evidence-based and conceptual arguments avoids conflating established findings with speculative, yet ethically significant, concerns. This anticipatory and interdisciplinary literature survey further attempts to identify future research directions, which can then inform the development of evidence-based recommendations and support a nuanced, ethically informed use of VR in psychiatric care. In the first step, we will describe what types of potential risks are associated with XR (Chapter 3). In a second step, we will illustrate the relevant vulnerabilities of psychiatric patients. We capture these vulnerabilities by means of specific symptoms of psychiatric disorders. We focus on symptoms rather than conditions to be more specific in detecting patients at risk (Table 1) – thus applying a transdiagnostic approach (Chapter 4). In a final step, based on this analysis, we formulate recommendations that should be considered in accordance with the principle of *primum non nocere* (Chapter 5). By taking a close look at the potential risks of VR therapies in psychiatry, we want to contribute to the safe implementation of this promising new medium and, in the spirit of the editors of the Lancet 1991, prevent promising therapies from being withheld from patients due to a failed risk assessment^15^.

**Table 1:** XR associated vulnerabilities of psychiatric conditions

Symptom/Syndrome	Risk	Vulnerabilities	Mitigation Strategies
DEREALISATION	Dissociation	PTSD, personality d isorders, depersonalization and derealisation syndrom	Time-limited exposure, pre- and post therapeutic debriefing
DEPERSONALIZATION	Dissociation	PTSD, personality disorders, depersonalization and derealisation syndrom	Time-limited exposure, pre- and post therapeutic debriefing
HALLUCINATIONS	Blurring boundaries of reality	Schizophrenia, psychotic disorders, dementia	Pre- and post therapeutic debriefing
DELUSIONS	Exacerbation of pre-existing psychotic symptoms	Schizophrenia, psychotic disorders	Pre- and post therapeutic debriefing
DELUSIONS OF CONTROL	Exacerbation of pre-existing psychotic symptoms, *Proteus Effect*	Schizophrenia, psychotic disorders	Pre- and post- therapeutic debriefing, possible *Proteus Effect*
THOUGHT INSERTIONS	Exacerbation of pre-existing psychotic symptoms, *Proteus Effect*	Schizophrenia, psychotic disorders	Pre- and post therapeutic debriefing, possible *Proteus Effect*
TRAUMATISATION	Re-traumatisation	Previous experiences (e.g., violence, abuse, neglect)	Pre- and post- therapeutic debriefing, limited exposure
ANXIETY	Exacerbation of pre-existing symptoms	Anxiety and phobic Disorders, PTSD, cybersickness, OCD	Pre- and post- therapeutic debriefing, assisted exposure based on cognitive behavioral therapy concepts (e.g., anxiety increases during exposure and exposure continues until anxiety decreases)
COGNITIVE IMPAIRMENT AND DECLINE	Cognitive overload, blurring boundaries of reality	Dementia, schizophrenia	Assisted and time-limited exposure
SOCIAL WITHDRAWAL	Social isolation	Anxiety disorders, ASD, depression	Ecological interventions that promote socializing

Our review follows the quality criteria as proposed in the quality assessment scale SANRA “to contribute to improving the standard of non-systeematic reviews". SANRA comprises the criteria “(1) justification of the article´s importance to the readership, (2) statement of concrete aims or formulations of questions, (3) description of the literature search, (4) referencing, (5) scientific reasoning, (6) appropriate presentation of data”^16^ We argue that we are fulfilling these aforementioned basic requirements as requested for a narrative review.

### Real risks of virtual experiences

In a recent article, Slater proposes that the power of immersive systems arises from four experiential illusions that give XR its psychological impact: 1. The illusion of embodiment, 2. the place illusion, 3. the plausibility illusion and 4. the illusion of being with others in the virtual environment^17^. While the notion of illusions to describe the experiences of being embodied at a specific place with others has recently been challenged since “any subjective perception and experience, any qualia, must be assumed as real”^18^, the resulting effects nevertheless make XR a powerful tool for psychiatric and psychotherapeutic applications, but at the same time, introduce ethical concerns. For one, there are still general technology-related risks of VR like cybersickness^19^. Cybersickness is a phenomenon that can occur during movements in virtual worlds and can lead to exhaustion, dizziness, nausea, and even vomiting. It is assumed that this is due to a conflict between vestibular and visual informations, e.g., in cases when we are not actually moving, but the visual impression leads us to believe that we are moving. While technological advancements seem to have mitigated these side effects, the general problem persists and still requires careful system design, proper evaluations, and appropriate countermeasures^20^. Additionally, the immersive properties can also cause unforeseen and unexpected results, i.e., on the user’s decision making processes^21,22^, also calling for careful designs and evaluations. In the following, we describe specific risks related to virtual embodiment (3.1), the possibility of interacting socially in virtual environments (3.2) and the virtual environment itself (3.3).

### The virtual body

XR can influence the self-concept of a person in different ways. A key factor in this regard is the experience of one´s own body or the virtual embodiment, which can correspond to the real body but by no means has to. Virtual embodiment can open up many different possibilities for a user. For example, it is possible to virtually slip into completely different bodies (different appearance, gender, species)^23^. However, a virtual body can also be harmed in various ways. The virtual embodiment as a representation of a person establishes a relationship between real persons and their virtual experiences. Consequently, virtual representations influence our experiences. A key example is the so-called *Proteus effect* in this context^24^. This describes the effect that the alteration of one’s own bodily virtual representation can substantially change the behavior of that person in VR and even beyond the virtual exposure^25,26^. First evidence suggests that a similar effect can be elicited also by other persons’ virtual characters^27^. Notably, the *Proteus effect* persists for some time and can even be experienced after the virtual experience has finished. Therapeutically, this is potentially very relevant.

However, this effect can be understood not only as a modification but also as manipulation of behavior. The risk of manipulation arises if the *Proteus Effect* is not explained transparently to the user but is used by the therapist aiming at a change of behavior unbeknownst to the user. Of course, the main goal of behavioral therapies is to change feelings and thoughts through the change of behavior, however, it is usually a conscious and transparent process that is communicated between patient and therapist and always leaves room for the autonomous decision-making of the patient. From an ethical perspective, two aspects are relevant here: Firstly, the possible feeling of being influenced by someone follows a paternalistic attitude. In particular, patients suffering from so-called ego disturbances as part of a psychotic disorder could react anxiously or even experience an exaggeration of their symptoms when they feel they are being manipulated by others. Patients with symptoms of thought insertions and delusions of control appear to be especially at risk here because, in the worst case, the *Proteus effect* can lead to unconscious behavior changes through which the patient could possibly see the external influence confirmed. Secondly, if that patient perceives an altered virtual representation as negative, it could lead to unfavorable changes in reality, expressed as reduced self-confidence. The perception can be induced by the representation itself or by the reaction of independent observers. A significant indication that points in this direction is the relevance of social interactions in XR. Banakou and colleagues were able to show that although the implicit racial bias of white individuals decreased after they were virtually embodied in a black body, it increased again when this virtual body was evaluated negatively by a group of virtual characters^28^. As a positive reaction, self-observation of a virtual body-double engaged in positive social interaction reduces persecutory thoughts^29^.

Virtual embodiment is also discussed in another context, namely it can be potentially used as an opportunity to develop empathy for certain groups by providing the opportunity to take their perspective. Specifically, this is discussed for offenders (e.g., in the context of domestic violence), who could put themselves in the position of their victims^30^. The goal is “to alter the way of thinking of a subject”^31^. This approach is justified by the fact that VR creates convincing experiences while at the same time being “not real”. Ethically, it will be debatable whether – and if so, to what extent – the simulation of a violent experience, as an example, is permissible and proportionate to the potential to change the behavior of the offender. The more convincing a virtual experience of violence is, the more likely it is that a person who experiences it will react psychologically as if it was a real experience. There are ethical positions that demand that what is not allowed to be done in reality should not be allowed in virtual environments either^32^. In VR, there are examples of violation against virtual bodies by third parties, such as undressing an avatar that appears naked thereafter or undergoes sexual harassment, which has been shown to have a severe negative psychological impact on users^33^.

Finally, virtual bodies can also be reliable replications of the users in real life^34,35^. The personalization of avatars has raised interest also in the context of therapeutic interventions. They can become potentially useful applications as experiences covering body ownership, presence, emotional response^35^, or body awareness, can be improved. For example, these effects have recently been explored in the context of therapies to treat obesity disorders^36,37^ and seem generally to be able to manipulate one’s body image^38^, opening-up a much wider scope of potential therapeutic applications. There are also applications for the treatment of anorexia nervosa, which are based on the assumption that distorted multisensory integration plays a central role in eating disorders. Here, various approaches aim to correct dysfunctional body perceptions^39^. The possibilities of different body perceptions in various virtual bodies and their perceptions from first-person and third-person perspectives significantly expand the therapeutic spectrum and are only possible within the virtual context.

However, personalized avatars induce additional risks of potential avatar theft and fraud^33^, with far-reaching consequences ranging from authenticity^40^ and trust^41^ to the question of appropriate^42^ countermeasures^43^. These identity-related risks have not only psychological, but also justice dimensions, as individuals with fewer digital literacy skills or resources may be disproportionately exposed. These risks lead over to the next section, as they concern both the virtual body and the forthcoming discussion of virtual social interaction.

### Real virtual social interactions

It is a very elementary relationship with others that is established by seeing and being seen^44^. Across diverse mental health conditions and settings, convergent evidence shows that social support—both perceived and received—improves clinical outcomes, buffers stress, and can reduce relapse risk^45,46^, but unfortunately, social support is often limited in the context of mental illness. Some developments appear to be very promising in supporting patients in this regard. Realistic applications, in the sense of training platforms^47^, offer the opportunity to practice social interaction at various levels of difficulty in the sense of VRET. Especially for people with social anxiety disorder, these programs can be a valuable opportunity to gradually improve their ability to deal with anxiety in real life through virtual training^48^. Patients with social anxiety fear negative evaluation by their fellow human beings, which ultimately leads to avoidance of social situations and interactions. Due to natural circumstances, a gradual increase in the level of intensity is difficult and not easily controllable in in-vivo exposure. VRET offers significant advantages in this regard. However, many other mental conditions are also associated with various forms of anxiety including but not limited to social anxiety, and some can also benefit from virtual training situations.

Besides the various options of virtual training of real-life situations, it is also possible to modify a person’s non-verbal behavior, such as gestures and eye movements, in real-time in VR. Prototypes of system architectures through which nonverbal behavior can be modified in real-time already exist^49,50^. This was referred to as “social augmentation” and could be, for example, helpful when people with different communication behaviour interact with each other, who, due to their different communication styles, do not share the same non-verbal behavior, such as persons with autism spectrum disorder (ASD)^49^. For persons with ASD, social augmentation opens up the possibility of a non-verbal interaction similar to that of persons without ASD. However, suppose that people who suffer from significant limitations in social interaction competences in reality are unintentionally unable to comply with social norms on a non-verbal level and thus also send misleading messages in social interactions. In that case, patients may perceive this augmentation tool as a benefit but may also maintain the fear of interacting socially because the limitations probably persist in reality. As a consequence, a withdrawal from reality into the virtual world seems possible and could potentially result in a loss of social participation^51^. On an individual level it is also conceivable that users lose their sense of control over their own actions if their own behaviour is modified in real-time. Consequently, so-called agential uncertainty could develop, namely the restriction or even loss of the experience to be the autonomous author of one´s own actions^8^. As platforms such as the Metaverse where people can meet represented by avatars become increasingly important, understanding social interactions in virtual environments and their meaning for a user’s self-concept is a high priority both for basic and applied research.

In this context, another phenomenon, identity infringement, also plays a significant role. It appears to be the most frequently expressed concern in the literature regarding personalized avatars^33^, as already introduced at the end of the last section in the context of the virtual body. The threat results from the fact that in social interactions, both parties are ascribed an identity. In an interaction, both parties intuitively assume that the other is who he or she claims to be. Under these conditions, communication and an exchange of information take place. Pretending to have a false identity can lead to the disclosure of confidential information in the belief that one is interacting with a trustworthy person, e.g., a therapist. In therapeutic contexts, the accountability of avatars is of particular importance^52^. However, it is not only the reliability of an avatar’s identity that poses a problem. There is also the risk that de-anonymization and disclosure of personal information develops based on an avatar’s appearance and behavior. In medical contexts, this could include all kinds of health-related information. Legally, these phenomena are gaining increased attention^53,54^.

### Virtual environment

Lastly, the concrete virtual environment provides immersive experiences in a simulated environment. In that context, convincing digital simulations of real physical environments make it possible to test changes and applications in a simulated space. However, despite any real-world resemblance, if the artificial stimuli match (are congruent to) the sensory and perceptual input humans receive from the physical world around them, these artificial inputs will generate psychological and physiological responses^18^. These responses in cases of congruency are the reason for the capacity of VR that it can act as an “experience machine”^55^ in which computer programs can systematically modify experiences. In the case of physical environments, the distinction seems clearer than in the case of human experiences through virtual embodiment, social interactions in XR and virtual behavior modifications. In the case of physical environments, the quality of the psychological experience in virtuality is similar to that of reality in terms of its phenomenological content. In the psychiatric context, there are patients who, due to their condition, have difficulties making this distinction between the real and the virtual world. The boundaries can, therefore, become blurred. The fear of this loss of boundaries and how to deal with it is a real concern for patients. This issue has consequences for informed consent, too. Participants are either confused already from the start of the VR visit or they may lose track at some point. The question arises whether an informed consent that was obtained before confusion or misunderstanding arise is still valid. This leads to the question under which conditions do we require an “ongoing” and “repeated” informed consent, and in which cases do we allow initial consent to persist through such a period of incapacity to differentiate reality from simulation? In the context of XR, questions concerning reality and the perception of reality thus, for the first time, have become a crucial subject of medical ethics^9^.

Associated with the concern of blurring boundaries is the fear that derealisation and depersonalisation phenomena may occur. *Derealization* is defined as “experiences of unreality or detachment with respect to surroundings”; depersonalisation refers to ‘experiences of unreality, detachment, or being an outside observer with respect to thoughts, feelings, sensations, body or actions’^56^. But in contrast to blurring boundaries reality-testing remains intact during a *derealization* or *depersonalisation* experiences^56^.

The concern that such phenomena could occur results from anecdotal reports from the gaming industry. Here, it is reported that users have experienced *depersonalization* after prolonged XR exposure. So far, there is no empirical evidence, but it is currently subject of research^57^. However, it is reasonable to assume that patients who experience *depersonalization* and *derealization* in the context of their underlying psychiatric condition are at higher risk of developing it after XR exposure.

### Who is at risk of getting harmed?

This section seeks to provide some considerations on which patient groups might exhibit increased vulnerability and thus should therefore be monitored particularly carefully during medical XR interventions (or, perhaps, in some cases, should not receive XR treatment at all)^58^. Beyond individual psychopathological factors, vulnerability in XR medicine also has structural dimensions. Access to secure, supervised XR environments presupposes technical literacy, economic means, and institutional safeguards. These preconditions create structural vulnerabilities that can amplify inequities in who benefits from or is harmed by XR interventions. To date, there is no empirical evidence for the following considerations available so that we are forced to speculate. In the following, partly based on the analysis so far, we focus on symptoms and syndromes as defined by DMS 5 rather than on diagnoses of mental disorders (Table 1). We are not differentiating between different age groups (e.g., children, adolescents, adults, elderly) as most of the symptoms and syndromes presented below can occur in all age ranges.

#### Derealization and depersonalisation

*Derealization* is defined as a syndrome of “experiences of unreality or detachment with respect to surroundings”. The experience of moving in a convincing virtual environment that allows for experiences and social interactions just like in the real world could intensify the feeling of being disconnected from the real world. *Depersonalisation* is defined as “experiences of unreality, detachment, or being an outside observer with respect to thoughts, feelings, sensations, body or actions”. Being virtually embodied repeatedly or for an extended period of time may lead to an exacerbation of experiences such as detachment or being an outside observer with respect to thoughts, feelings, sensations, body or actions in reality. During a *derealization* or *depersonalisation* experience reality-testing, however, still remains intact.

#### Delusions

*Delusions* refer to “fixed beliefs that are not amenable in the light of conflicting evidence”. For instance, *persecutory delusions* describe the belief that someone is being harmed or harassed by an individual or a group. Some patients hold the belief of being spied on. Patients who are haunted by the fear of being followed and/or spied on may feel particularly insecure about a possible theft of a wide range of personal data. In case of a virtual therapist, the uncertainty as to whether the person of the therapist is really who he or she claims to be also potentially becomes a particular threat to these patients. Another relevant phenomenon is the symptom of *thought insertion* which belongs to the group of bizarre delusions. A delusion is “bizarre” when the experience of control over one´s own mind or body is lost. The conviction that thoughts have been sent or broadcasted into a patient’s mind by someone else defines the symptom of thought insertion. Patients suffering from thought insertion and experiencing their own behavior as having changed - possibly as a result of a *Proteus effect* - could attribute this change to thoughts or intentions-to-act being sent from other persons or agents to manipulate their behavior. This could result in an exacerbation of the symptoms. A particular type of bizarre delusions are *delusions of control* referring to the conviction that one’s body or actions are being acted on or manipulated by some outside force^56^. Patients who may already feel that they are being controlled and manipulated from outside, e.g., as part of a psychosis, may experience this more intensely as a result of a *Proteus effect*.

#### Hallucinations

*Hallucinations* are “perception-like experiences that occur without a real external stimulus. They are vivid and clear, with the full force and impact of normal perceptions, and not under voluntary control”. Any modality of sensory perception can be affected^56^. Virtual environments also show some characteristics of *hallucinations* as they convey convincing sensory impressions (at least visually and auditorily). Whereas participants should be able to clearly differentiate whether the experience is a simulation in VR or the real world around them, patients who already suffer from *hallucinations* could be overwhelmed and irritated by even more false sensory impressions. As a result, it is conceivable that patients may have difficulties with reality testing.

#### Traumatisation

Certain scenarios may “reactivate” traumatic experiences. For instance, above, we mentioned the possibility of XR for domestic violence. However, some of the people who would be candidates for receiving such treatment may be traumatised themselves, having, for instance, experienced aggression in their childhood or later in life.

#### Anxiety

Anxiety disorders are among the most common psychiatric disorders worldwide and are characterized by uncontrollable worries, fears, and hyperarousal^47^. In addition, many other mental conditions are associated with symptoms of anxiety (including disorders like major depressive disorder, psychosis, obsessive compulsive disorder ( = OCD)). Although the triggers vary, the phenomenological experience of anxiety can be comparable to symptoms of primary anxiety disorders. Many forms can be treated psychotherapeutically using exposure therapies. The advantages of VRET are manifold: for example, numerous repetitions and control over the level of difficulty are possible. Nevertheless, due to its realistic nature, VRET can also cause patients to feel overwhelmed by the confrontation with anxiety-inducing stimuli. A worsening of anxiety and panic symptoms is conceivable.

Further, anxiety can often be associated with or result from physical symptoms such as increased heart rate, sweating, dizziness, shortness of breath, trembling, nausea, and others. Patients often experience these symptoms as very threatening. Some of these symptoms can also occur with cybersickness. It is conceivable that patients react to these physical symptoms, triggered by cybersickness, with particular sensitivity and increased anxiety, as they are familiar with them from the context of anxiety.

#### Cognitive decline

The decline or impairment of cognitive functions can lead to a lack of distinction between real and virtual environments. It can be speculated that the more the virtual environment resembles reality, the more difficult it is for the patient to distinguish between the two. The well-known phenomenon of “willing suspension of disbelief”^59^ may not be reversible for individuals with the aforementioned symptoms.

#### Social withdrawal in the real world

Patients, who have difficulties interacting socially with others, such as those with ASD or anxiety disorders, particularly social phobia, could use XR and its modification options to interact “normally”. Social interactions in the real world could be reduced further and further due to discomfort or fear of rejection.

### Primum non nocere: recommendations for how to prevent harm

#### General aspects

The developments of designs of VR applications must obviously take into account the state-of-the-art in technological and methodological advances in the field, for instance, with respect to novel achievements that become possible with new techniques but also side effects like cybersickness. A continuous observation of the scientific literature allows to start any new XR application with a proper analysis of potential risks in this quickly advancing field. Any new technology needs to test and evaluate effects during examinations of healthy users first. Early testing should also include suitable and informative technical measures. Evaluations should include not only objective measures but also general usability and user experience measures in addition to reliable measures for the specific target effects. An appropriate unobtrusive monitoring focusing on unwanted effects should be implemented where possible.

#### Ensure data security and protect digital identities

One key technical requirement for the responsible diagnostic and therapeutic use of XR is the adequate understanding of and information about the extent to which data collected through XR exposure can be secured at all or are private. Since any behavioral data (e.g., eye tracking, social interaction, orientation) can be collected in XR, which may also allow conclusions to be drawn about disease risks, it must be clear to a patient what can and what cannot be protected. A patient’s digital identity must also be protected against misuse, identity theft, and manipulation.

#### Informed consent

Informed consent is one of the fundamental ethical requirements for a trustworthy relationship between therapist and patient. So far, there is no standard approach to informing patients about XR interventions or, rather, which particular aspects must be discussed before XR applications. In general, informed consent in XR therapy is not a one-time act but a relational and situated process shaped by trust, comprehension, and context. Recognizing consent as relational highlights the need for iterative, dialogical communication, particularly for patients whose vulnerability may be cognitive, emotional, or social.

Like other exposure therapies, VRET may cause distress to the patient, although *desensitization* through continued exposure is often the objective. This can induce a particular tension. Generally, patients are entitled to withdraw consent at any time, and therapy should be stopped unless abrupt discontinuation poses a risk of harm to the patient. In these cases, breaks to debrief prior to re-entering the XR scenario could be helpful. In other cases, a more challenging problem for informed consent occurs where a patient appears to have become confused about reality while in XR. The informed consent process will have informed the patient of this possibility. Still, the patient’s responses in XR could suggest that they have gone beyond “willing suspension of disbelief” and are confusing XR and reality. If this happens, they may no longer be fully competent from a decision-making perspective and unable to choose whether or not to continue in XR. In these scenarios, physicians should monitor carefully for signs of severe distress and discontinue the therapy where needed. If therapeutically beneficial, continued treatment under these circumstances could be discussed ahead of time with the patient and with a substitute decision-maker who could step in and re-iterate consent on behalf of the patient (if this is a possibility regarding such an intervention in the relevant national jurisdiction). However, in principle, if doubt arises about the patient’s consent during XR therapy, treatment should be stopped.

#### Transparent communications: who is virtually involved?

For a patient to trust a therapeutic intervention, it is necessary to be aware of who is behind the virtual representatives with whom they interact in case of virtual social interactions. Above all, deception through misleading virtual representations (a possibly photorealistic avatar looks like the treating psychotherapist but is actually someone else) must be avoided. It must also be communicated transparently if the interaction involves digital characters, i.e., program-driven interaction partners.

#### Maintain autonomy, discuss possible behavioral modifications in detail before and after

The desired behavioral modifications must be explained to the patient. If, for example, a *Proteus effect* is to be used therapeutically, this must be communicated transparently, especially if post-interventional changes are also expected or even desired. But even for behavioral modifications that “only” apply to XR, such as the social enhancement of non-verbal behavior described above, it is important to provide a solid preparation to prevent feelings such as agential uncertainty or the exacerbation of psychotic symptoms.

#### No knowledge about long-term and unintended adverse effects

As is often the case with new technologies, there is limited knowledge about long-term and unintended adverse effects. This, too, must be communicated transparently.

#### Screening for vulnerable patients

Screening for specific vulnerabilities (e.g., in terms of traumata or phobias) that may be relevant for an XR scenario in a psychiatric context is vital to prevent the exacerbation of existing symptoms through careful monitoring and follow-up. The screening should only be used to identify patients who require more intensive support and not to exclude them from XR applications. Recognizing the symptoms described in Chapter 3 can serve as a starting point. Screening should also consider contextual vulnerabilities such as social and economic marginalization, limited digital literacy, or negative prior experiences with the applied technology. Such broader awareness helps prevent the inadvertent exclusion of already underserved patient groups.

#### Participating patients in the development of clinical applications

Just as there are many difficulties in the development and implementation of a new technology in the treatment concepts for patients, there are also many opportunities. There are increasing calls to involve future users in the development process in order to increase acceptance and compliance in the follow-up^58^. Such an approach also enables a better understanding of concerns. Particularly in the field of virtual interaction, empirical research should take into account the preferences and concerns of patients in advance of the development process to prevent adverse outcomes^58^. Beyond consultation, co-creation frameworks can treat patients as epistemic partners. Especially in psychiatry, participatory designs help surface *tacit* vulnerabilities and trust dynamics that are often invisible to developers or clinicians. Besides this user-centered development process, which necessarily requires the involvement of patients, all other relevant potential stakeholders (patients relatives, therapists, computer scientists) should be considered as well^60^.

#### Clinical training

Finally, the safe and ethical use of XR requires adequate training of the involved clinicians. Therapists and technical staff should be particularly educated to recognize distress, understand XR-specific consent challenges, and navigate digital identity and privacy issues. Therefore, ethical literacy should be explicitly included in XR training curricula.

## Conclusion

Current developments show that XR will be an important enrichment for diagnostic and therapeutic applications for psychiatric conditions in the future. In order to use these XR tools as efficiently and safely as possible, it is crucial to examine the ethical implications and risks for patients. In this article, we tried to explore both based on the oldest principle of medical ethics, namely *primum non nocere* or the principle of non-maleficence. We have not only described potential risks but also, in a first attempt, related them to the specific vulnerabilities of psychopathological symptoms. In doing so, we explicitly do not pursue the goal of excluding these vulnerable patients from the opportunities that XR offers in a clinical context but rather describe means to identify patients whose XR exposure must be done with increased vigilance and care in a differential ethics framework for XR applications in psychiatry. As XR becomes increasingly integrated into psychiatric care, ethical guidance must also consider systemic justice. Issues of equitable access, resource distribution, and protection from digital divides are central to realizing non-maleficience for all patients. Future research should empirically investigate the increased risks of vulnerable populations as speculated here. In addition, with the increasing establishment of virtual clinical applications, it is necessary to develop guidelines for the use of XR in an interdisciplinary exchange between technical developers, clinical practitioners, patients and ethicists.

## Data Availability

No datasets were generated or analysed during the current study.
